# Assessment of acceptance and associated factors of HIV pre-exposure prophylaxis among commercial female sex workers in drop-in centers selected sub-cities of Addis Ababa, Ethiopia

**DOI:** 10.3389/fpubh.2024.1462648

**Published:** 2024-11-29

**Authors:** Trhas Tadesse Berhe, Elefie Asfaw Asfaw, Getachew Weldyohanes Tedla

**Affiliations:** Department of Public Health, Yekatit 12 Hospital Medical College, Addis Ababa, Ethiopia

**Keywords:** acceptance, Addis Ababa, Ethiopia, female sex worker, HIV, pre-exposure prophylaxis

## Abstract

**Background:**

Globally, female sex workers (FSWs) face high risk of HIV, particularly in regions like sub-Saharan Africa. In Ethiopia and Addis Ababa, the impact is significant. Implementing WHO-recommended measures, such as pre-exposure prophylaxis (PrEP), is crucial to reducing new HIV infections and addressing service access disparities among FSWs. Thus this study aimed to assess the acceptance of Pre-Exposure Prophylaxis (PrEP) among commercial female sex workers in selected sub-cities of Addis Ababa, Ethiopia, 2022.

**Method:**

Institution-based cross-sectional study design was conducted on three randomly selected sub-cities of Addis Ababa from June 20 to July 30, 2022. All (358) commercial sex workers available during the study period were included. A structured, pretested, and interviewer-administered questionnaire was used to collect the data. Logistic regression was used to identify factors associated with acceptance of pre-exposure prophylaxis and statistical significance was determined at *p*-value <0.05. An odds ratio with a 95% confidence interval was used to measure association estimates.

**Result:**

A total of 358 female sex workers responded, 67.9% (95% CI: 63.7, 73.2%) were willing to take pre-exposure prophylaxis. Acceptability of pre-exposure prophylaxis was significantly associated with the accessibility of pre-exposure prophylaxis at easily reachable areas (AOR3.786; 95%CI: 1.449, 9.894) and knowledge about pre-exposure prophylaxis (AOR 3.270; 95%CI: 1.336, 8.001).

**Conclusion:**

Acceptability of pre-exposure prophylaxis among female sex workers was 67.9% which is low. Accessibility of pre-exposure prophylaxis is an easily reachable area and knowledge of about it could significantly affect its acceptability.

## 1 Introduction

HIV remains a major public health concern, disproportionately affecting female sex workers (FSWs) worldwide (1). FSWs are among the most vulnerable populations due to factors such as inconsistent condom use, multiple sexual partners, and limited access to health services, which contribute to their heightened risk of HIV infection. In Ethiopia, this issue is particularly concerning. A systematic review conducted in 2019 estimated that there are approximately 200,000 FSWs in the country, with the majority (57.5%) being 24 years old or younger, and 14% aged 19 or younger (2). This indicates that a significant number of FSWs are in the younger age group, highliting the urgency to address HIV prevention strategies for this population. Addis Ababa hosts one of the largest populations of female sex workers (FSWs) in Ethiopia, significantly exceeding the numbers found in other regions (3). The impact of HIV remains severe, especially among FSWs, with the highest prevalence observed in this group (4). According to studies done by the Central Statistics Agency and IMF in 2016, HIV prevalence among the general population in Ethiopia ranges from 0.8 to 4.8% (5). A national surveillance study on Most Risk Populations revealed a significantly higher HIV prevalence of 23.8% among female sex workers in all regional capitals (6). Female sex workers (FSWs) globally face a substantial HIV burden and encounter challenges in accessing health services, particularly pronounced in high-prevalence regions like sub-Saharan Africa (7). Despite declines in HIV prevalence, the 2016 Global AIDS update reveals persistently high rates among FSWs (8). They are considered a hard-to-reach population, with limited uptake of HIV services in Ethiopia, prompting a recognized need for targeted interventions (9, 10). Ethiopia, in alignment with global goals, aims to reduce new HIV infections and end AIDS as a public health threat by 2030, as reflected in national strategies (1, 6). Evidence suggested comprehensive HIV prevention for sex workers, led by their communities, is crucial (11, 12). The recent introduction of pre-exposure prophylaxis (PrEP) for high-risk populations, including female commercial sex workers, highlights the importance of adherence and acceptability for its effectiveness in reducing new HIV infections. Ethiopia is actively working on preventive measures to align with the global goal of ending AIDS by 2030, particularly focusing on the well-being of female sex workers and sero-discordant HIV-negative individuals (1, 4, 6).

Various studies among different populations, including female sex workers (FSWs), reveal substantial interest in PrEP. Participants in Guangxi, China, expressed willingness if proven safe and effective, while studies in Dar es Salaam, Tanzania, Harare, Zimbabwe, and Kolkata, India, demonstrated high acceptance rates, especially if PrEP was provided free of cost (13–16).

Studies in a high-risk Peruvian population and Zambia and Uganda emphasized factors impacting PrEP use, such as out-of-pocket costs, efficacy, potential side effects, and stigma (17, 18). WHO recommends offering PrEP to those at substantial risk, making it crucial for FSWs’ needs to be addressed to achieve Ethiopia’s 2030 HIV/AIDS elimination goal (19).

One such prevention strategy is the use of pre-exposure prophylaxis (PrEP), which has proven highly effective in reducing HIV transmission among high-risk groups, including FSWs (8). However, for PrEP to be successful, high levels of awareness, acceptability, and adherence are required (20, 21). While studies in other countries such as China, Tanzania, Zimbabwe, and India have shown substantial interest in and acceptance of PrEP among FSWs (13–16), research on the awareness, perceptions, and uptake of PrEP among FSWs in Ethiopia remains limited.

This study aims to fill this gap by specifically assessing the acceptance and associated factors influencing PrEP uptake among FSWs in Addis Ababa. Unlike previous studies that focused on broader or different populations, this research centers exclusively on FSWs in drop-in centers, offering new insights into their attitudes and behaviors regarding PrEP use. The findings from this study will provide critical baseline data for future interventions and policy formulation, supporting Ethiopia’s national strategy to reduce new HIV infections among key populations.

## 2 Methods and materials

### 2.1 Study area and period

The study was conducted at Drop-in Centers (DICs) in Addis Ababa, Ethiopia, from June 20 to July 30, 2022. Addis Ababa, the capital city of Ethiopia and a major diplomatic hub in Africa, is divided into 11 sub-cities and hosts 12 Drop-in Centers. These centers play a critical role in supporting key populations, such as female sex workers, by providing essential HIV prevention and treatment services. Addis Ababa, as the largest city in Ethiopia, is a key destination for migrants from other regions, which contributes to the high number of female sex workers. The outflow of people, combined with the city’s economic opportunities and its role as a tourist destination, further increases the demand for commercial sex work.

The selected sub-cities for the study Akaki, Yeka, and Kirko are densely populated and represent areas with diverse socio-economic dynamics, including significant migrant populations. As of 2024, the population of Addis Ababa was estimated at 5.7 million, growing annually at 4.45%, which accounts for approximately 20% of Ethiopia’s urban population (22). Estimates show that the city has one of the highest concentrations of female sex workers in Ethiopia (23). The study specifically focused on female sex workers selected from Drop-in Centers located in these three sub-cities.

In terms of HIV prevention, diagnosis, and treatment, Addis Ababa has a well-established healthcare infrastructure. The Drop-in Centers provide community-based comprehensive HIV services, offering essential support to female sex workers. Services include free condoms, HIV testing, counseling, antiretroviral therapy (ART), and other sexual and reproductive health services. In addition to the DICs, numerous public health facilities, such as health centers and hospitals, are available across the city to provide further HIV care and treatment. Both the government and non-governmental organizations are actively working to reduce HIV transmission through broad preventive measures and improved access to healthcare services.

### 2.2 Study design

Institution-based cross-sectional study design was conducted to assess the acceptance of pre-exposure prophylaxis among female sex workers coming to drop-in centers in Addis Ababa, Ethiopia, 2022.

### 2.3 Source of population and study population

The study population consisted of all female commercial sex workers who visited the drop-in centers in the selected sub-cities of Addis Ababa, Ethiopia, during the study period for various reasons. Given the limited number of female sex workers accessing these centers, we employed a census approach rather than a sample size calculation, which allowed us to include all 358 individuals who met the inclusion criteria.

### 2.4 Eligibility criteria

This study included female sex workers residing in the selected sub-cities of Addis Ababa during the data collection period, who were HIV-negative at that time, eligible for PrEP, and willingly provided consent to participate.

### 2.5 Variables

The acceptability of PrEP was the dependent variable.

Commercial female sex workers’ awareness, Accessibility of PrEP, FSW knowledge about PrEP, FSW Stigma, and discrimination, socio-demographic characteristics like age, marital status, educational status, place of birth, income, engaging in sex work before arriving in Addis Ababa, total years of experience, in engaging in sex work, reasons for becoming a sex worker, substance use, alcohol use, and condom use were Independent variables.

### 2.6 Data collection tool and procedure

Structured interviewer-administered questionnaire was developed to collect socio-demographic, socioeconomic, and clinical characteristics of female sex workers (FSWs) in Addis Ababa. This questionnaire was designed by integrating relevant questions from established guidelines and previous literature (23–25).

To ensure the reliability and validity of the instrument, a pretest was conducted with a sample representing 5% of the target population and based on the feedback the questioner was modified. Training was given for data collectors and supervisors for 1 day on the objective, significance of the study, data collection tool, procedures and detailed instructions on how to administer the questionnaire effectively. To facilitate the recruitment process, collaboration was established with health providers at the Drop-In Center (DIC), ensuring that the data collectors were familiar with the study environment and the target population.

Female sex workers identified as HIV-negative and eligible for pre-exposure prophylaxis (PrEP) were recruited during the data collection phase. Informed consent was obtained from each participant prior to their inclusion in the study, provided that they understood the purpose of the research and their rights as participants.

The data collection process was supervised by the principal investigator along with two field supervisors, who monitored the data collectors to maintain adherence to the study protocol and ethical standards throughout the duration of the study.

### 2.7 Data analysis

The collected data were entered into Epi-data version 3.1 to reduce entry errors and avoid inappropriate values by fixing the range of values coded in the software. The data were analyzed using SPSS version 23. For the first objective, descriptive statistics were used, and findings were presented in a frequency table. For the second objective, which focused on identifying associated factors influencing PrEP acceptance, binary logistic regression analysis was performed. The selection of independent variables was guided by theoretical frameworks and empirical evidence from existing literature on HIV prevention, specifically tailored to the context of commercial female sex workers. Initially, each independent variable was assessed in a bivariate analysis with the dependent variable (PrEP acceptance), and those with a *p*-value <0.25 were considered for inclusion in the multivariate logistic regression model. In the multivariate analysis, variables with a p-value ≤0.05 were deemed statistically significant. To ensure robustness, criteria such as the Akaike Information Criterion (AIC) and Variance Inflation Factor (VIF) were utilized to check for multicollinearity, with a VIF value greater than 10 indicating high collinearity. The Wald test was employed to assess the significance of each predictor, ensuring that only statistically significant variables contributed to the final model. Adjusted Odds Ratios (AOR) with a 95% Confidence Interval (CI) were reported.

### 2.8 Data quality management

Before data collection the questionnaire was translated into the local language Amharic and then back into English to ensure consistency. Before data collection, the questionnaire was pre-tested on 5% of the total study population at Bole sub-city. During data collection: the data collection and interviewing mechanism was strictly supervised throughout the data collection period by the assigned supervisors and the principal investigator. Questionnaires were checked for completeness and consistency at the site of the data collection by the principal investigator.

### 2.9 Ethical approval and consent to participants

Prior to data collection, ethical clearance was obtained from Yekatit 12 Hospital Medical College (Ethical Clearance Number 399/2022) and subsequently submitted to the Addis Ababa Health Bureau. The Addis Ababa Health Bureau then issued support letters to the sub-cities of Yeka, Akaki Kality, and Kirkos, which in turn provided further support letters to the respective designated areas (Drop-in Centers). To ensure confidentiality, all data were anonymized by assigning unique codes to participants, and any personal identifiable information was removed during the data collection process. Data were stored securely in password-protected files, with access restricted to authorized research team members only. Additionally, all interviews were conducted in private settings to ensure participant confidentiality. Written and verbal informed consent was obtained from all participants, and they were assured of their right to withdraw at any stage without any consequences. The study procedures strictly adhered to the principles outlined in the Declaration of Helsinki.

### 2.10 Operational definition and measurements

#### 2.10.1 Commercial female sex worker

Commercial FSW” refers to women who engage in sex work primarily for financial remuneration. Participants were identified as commercial FSW based on their self-reported occupation status. A survey tool was employed that categorized employment as commercial sex work.

#### 2.10.2 Acceptability

This refers to the extent to which female sex workers were willing and able to use PrEP consistently and correctly. Participants were asked to report their intention using yes/no questions, such as “Would you be willing to start taking PrEP if it were offered to you?” and “Would you be willing to take PrEP daily as prescribed?” (16). Those who answered “yes” were scored as 1, indicating they accepted PrEP, while those who answered “no” were scored as 0, indicating they did not accept PrEP.

#### 2.10.3 PrEP accessibility

We assessed PrEP accessibility through a combination of self-reported factors, including physical proximity to health facilities that provided PrEP services, awareness of PrEP among female sex workers, and barriers to accessing PrEP, such as cost, stigma, and healthcare availability. This variable referred to the extent to which PrEP medication was accessible to individuals at a nearby location, whether the area was easily reachable, and whether the medication was available free of cost. Participants were asked to report on these aspects using yes/no questions, such as “Is there a healthcare facility offering PrEP medication near your location?,” “Is the location where you can get PrEP easily reachable for you?” Based on the World Health Organization’s recommendation that individuals should live within a 5 km radius of a health facility for optimal access (26), and “Is the PrEP medication available to you free of cost?” (24). Those who answered “yes” were scored as 1, indicating positive availability, while those who answered “no” were scored as 0, indicating negative availability. The data were collected via structured surveys and interviews with participants, and PrEP accessibility was categorized into different levels (Yes and NO).

#### 2.10.4 Educational status

In this study, the educational status of commercial sex workers was categorized based on established classifications from the Ethiopian Demographic and Health Survey (47). The categories are defined as follows: *Cannot Read and Write* refers to individuals who lack any literacy skills; *Can Read and Write* describes those who possess basic reading and writing abilities but have not completed any formal education; *Primary Level* includes individuals who have completed their primary education, typically covering grades 1 to 8; and *Secondary Level* encompasses those who have finished secondary education, which includes lower secondary (grades 9 and 10) and upper secondary (grades 11 and 12).

#### 2.10.5 Knowledge about PrEP prophylaxis

This refers to the extent to which individuals understand the purpose, effectiveness, and usage of Pre-Exposure Prophylaxis (PrEP) as a preventive measure against HIV infection. Participants were asked to report their knowledge using yes/no questions, such as “Do you know that PrEP is used to prevent HIV infection?,” “Are you aware that PrEP must be taken daily to be effective?,” and “Do you know that PrEP is recommended for people at high risk of HIV infection?” (24). Those who answered “yes” were scored as 1, indicating they had correct knowledge about PrEP, while those who answered “no” were scored as 0, indicating a lack of knowledge. Respondents who had answered ≥4 /7 labeled as knowledgeable and ≤ 3/7, not knowledgeable (16, 24, 25).

## 3 Results

### 3.1 Socio-demographic characteristics of the study participant

A total of 358 female sex workers were interviewed, resulting in a response rate of 98%. The median age of the participants was 28 years, with the majority belonging to the age group of 25–29 years (38.5%). In total, 69.3% of the study participants were born outside of Addis Ababa, while 30.7% were born in Addis Ababa. Among those born outside of Addis Ababa, only 6.5% had a previous history of sex work.

The majority of female sex workers had a primary level of education (33.2%), and most participants (47.2%) were not married. Additionally, 55.6% of participants came to Addis Ababa to work as house servants, while 17.7% came to visit relatives. Among all participants, 61% became female sex workers due to financial problems, followed by 21.5% who were influenced by their peers. Among the total participants, the majority (57%) had a monthly income of 3,001–6,000 ETB. Among all participants, the majority (34.1%) had been engaging in sex work for 0–3 years. Female sex workers born outside Addis Ababa began engaging in sex work at an earlier age (13 years) compared to those born in Addis Ababa (14 years). Their maximum age at the start of engaging in sex work was 44 years and the minimum was 13 years, but the mean age at the start was the same for both groups at 22.18 years (Table 1).

**Table 1:** Socio-demographic characteristics of commercial female sex workers in drop-in centers of Addis Ababa, Ethiopia, July 2022.

Demographics characteristic	Frequency (*n*)	Percentage (%)
Age group		
18–24	93	26.0
25–29	138	38.5
30–34	78	21.8
35–39	37	10.3
>40	12	3.4
Income		
≤1,000	19	5.3
1,001–3,000	118	33.0
3,001–6,000	204	57.0
>6,000	17	4.7
Education		
Cannot read and write	81	22.6
Can read and write	109	30.4
Primary level	119	33.2
Secondary level	49	13.7
Marital status		
Married	61	17.0
Never married	169	47.2
Separated	102	28.5
Other (widow and divorced)	26	7.2
Place of birth		
Addis Ababa	110	30.7
Out of Addis Ababa	248	69.3
Engaging in sex work before Addis Ababa (*N* = 248)		
Yes	16	4.5
No	232	64.8
Experience in commercial sex activities		
0–3 years	122	34.1
4–5 years	110	30.7
6–10 years	89	24.9
>10 years	37	10.3
Reason for coming to Addis Ababa (*n* = 248)		
House servant	138	55.7
Education	33	13.3
To visit my relatives	44	17.7
To work as FSW	25	10.1
Disagreement with those I live with	8	3.2
Reason for becoming a commercial sex worker		
Financial problems	219	61.2
Loss of parents	33	9.2
Peer pressure	77	21.5
Conflict with those I live with	20	5.6
Divorce	9	2.5

### 3.2 Assessment of awareness and accessibility of PrEp

Among the total interviewed clients (358), 294 (82%) heard about PrEP. Among these 294 female sex workers, 132 (45%) were informed about its advantages, 44 (15%) about how it could be taken, 29 (10%) about its accessibility, and 89 (30%) about all the above information.

From these 294 female sex workers, 172 (59%) received information from healthcare providers, 53 (18%) from community mobilizers, 40 (14%) from friends, 16 (5%) from media, and 13 (4%) from peer educators among all interviewed commercial female sex workers (358), 74.6% reported that they received PrEP in their nearest area. All of the female sex workers reported that they received it free of cost. Additionally, 72.9% of them said it is easily reachable. Among the total 358 female sex workers, 296 (82.7%) knew about pre-exposure prophylaxis (PrEP) (see Table 2).

**Table 2:** Commercial female sex workers’ awareness and source of information and accessibility of drop in centers about PrEP in drop-in centers in Addis Ababa, July 2022.

Awareness and accessibility	Frequency	Percentage
Heard about PrEP		
Yes	284	79%
No	74	21%
What do you hear about PrEP(n = 294)		
How it can be taken	44	15%
Its advantages	132	45%
Its accessibility	29	10%
All	89	30%
Source of information (*n* = 294)		
Media	16	5%
Health care provider	172	59%
Friends (PrEP user)	40	14%
Community-based mobilizer	53	18%
Peer educator	13	4%
Near to their home(5 km) (Drop-in center)		
Yes	262	74.6%
No	96	25.4%
Free		
Yes	358	100%
Reachable		
Yes	262	72.9%
No	96	27.1%
Knowledge about PrEp		
Not knowledgeable	62	17.3
Knowledgeable	296	82.7

### 3.3 Alcohol and substance use

Among all interviewed 358 female sex workers (FSWs), 274 (76.5%) had consumed alcohol. Of those who consumed alcohol, 52.8% had taken it during their last sexual intercourse, and 21.5% were intoxicated. Additionally, 64% of the total interviewed FSWs had used substances. Among the 229 substance users, 149 (65%) had chewed khat, 61 (27%) had used shisha, and the remaining 19 (8%) had used cigarettes (Table 3).

**Table 3:** Alcohol and substance use among female sex workers in drop-in centers of Addis Ababa, July 2022.

Alcohol and substance use	Frequency	Percentage
Alcohol consumption	274	76.5%
Alcohol consumption during last sex work	189	52.8%
Intoxicated during last sex work	77	21.5%
Substance use	229	64%
Type of substance used (*n* = 229)		
Khat	149	65%
Shisha	61	27%
Cigarette	19	8%

### 3.4 Acceptance of PrEP

Among the 358 interviewed female sex workers (FSWs), 243 (67.9%) [95% CI 63.7 to 73.2%] accepted pre-exposure prophylaxis (PrEP). Various reasons were reflected by those 115(32.1%) study participants who refused 34 (29.56%) cited fear of stigma and discrimination, 21 (18%) were concerned about drug side effects, 16 (13.9%) lacked information about the drug, 14 (12.1%) preferred using condoms, 9 (7.8%) believed God keeps them safe, 8 (6.95%) said they are HIV negative, 7 (6%) felt confident in their protective measures, and 6 (5.2%) mentioned other reasons (dislike of the drug, inconvenience of daily pills; Table 4).

**Table 4:** Commercial female sex workers’ acceptance of PrEP in drop-in centers, Addis Ababa, July 2022.

Acceptance	Frequency	Percentage
PrEP acceptance (*N* = 358)		
Yes	243	67.9
No	115	32.1
Reasons for not accepting PrEP (*N* = 115)		
Fear of stigma and discrimination	34	29.6
Concerns about drug side effects	21	18.3
Lack of information about the drug	16	13.9
Preference for using condoms	14	12.2
Reliance on religious belief (God keeps me)	9	7.8
Belief that being HIV-negative is sufficient	8	7
Confidence in personal protective measures	7	6.1
Other reasons (dislike of the drug, inconvenience of daily pills)	6	5.2

### 3.5 Factors associated with acceptability of pre-exposure prophylaxis

Using binary logistic regression, an association between the acceptance of pre-exposure prophylaxis (PrEP) and socio-demographic characteristics (age, marital status, educational level, place of birth, income), as well as other variables (easily reachable, heard about PrEP, knowledge about PrEP), were examined. Variables with a *p*-value less than 0.25 in the binary logistic regression were then included in a multivariable logistic regression model. After performing multivariable logistic regression, predictor variables with a p-value ≤0.05 were identified as significantly associated with PrEP acceptance.

The results indicated that there was a highly significant association between the ease of reachability of the PrEP service area and PrEP acceptance. Individuals who had easy access to PrEP services were 3.7 times more likely to accept PrEP compared to those who did not have easy access (AOR = 3.786, 95% CI: 1.50–9.56).

Moreover, a significant difference was observed between those who were knowledgeable about PrEP and those who were not. Those who knew about PrEP were 3.2 times more likely to accept PrEP than those who were not knowledgeable (AOR = 3.27, 95% CI: 1.30–8.23).

However, socio-demographic factors including age, marital status, educational level, place of birth, income, and having heard about PrEP, did not show a significant association with PrEP acceptance in the multivariable logistic regression model (Table 5).

**Table 5:** Associated factors for PrEP acceptance among sex workers at drop-in centers in Addis Ababa, July 2024.

Variable	Category	PrEP Acceptance (Yes)	PrEP Acceptance (No)	COR (95% CI)	AOR (95% CI)
Age					
	15–24	64	29	1.036 (0.78, 1.38)*	1.777 (1.12, 2.82)
	25–29	96	42	1.02 (0.72, 1.45)	2.308 (1.45, 3.67)
	30–34	54	24	0.595 (0.35, 1.01)	2.083 (1.12, 3.88)
	35–39	21	16	0.906 (0.48, 1.71)	1.258 (0.54, 2.91)
	≥40	8	4		
Education					
	Cannot read and write	53	28	1.687 (0.97, 2.93)*	1.231 (0.53, 2.85)
	Can read and write	83	26	0.934 (0.53, 1.65)	1.602 (0.73, 3.50)
	Primary level	76	43	0.91 (0.52, 1.57)*	0.85 (0.40, 1.81)
	Secondary level	31	18	1	1
Marital status					
	Married	37	24	1.589 (0.89, 2.82)*	1.496 (0.66, 3.37)
	Never married	120	49	1.297 (0.80, 2.10)	1.688 (0.80, 3.56)
	Widowed	12	5	1.557 (0.52, 4.67)*	1.741 (0.42, 7.15)
	Divorced	6	3	1	1
Experience					
	0–3	87	35	0.939 (0.57, 1.55)	0.792 (0.38, 1.64)
	4–5	77	33	0.753 (0.43, 1.31)*	0.524 (0.23, 1.18)
	6–10	58	31	0.528 (0.29, 0.96)	0.408 (0.16, 1.03)
	>10	21	16	1	1
Income					
	>1,000	18	1	0.076 (0.01, 0.54)*	1.496 (0.66, 3.37)
	1,001–3,000	68	50	0.137 (0.08, 0.24)	1.688 (0.80, 3.56)
	3,001–6,000	145	59	0.133 (0.08, 0.22)	1.741 (0.42, 7.15)
	>6,000	12	5	1	1
Heard about PrEP					
	Yes	208	76	1.596 (0.95, 2.68)	0.279 (0.11, 0.72)
	No	35	39	1	1
Source of awareness					
	Media	9	7	1.898 (0.69, 5.20)	0.496 (0.13, 1.89)
	Provider	122	50	2.051 (1.25, 3.38)	1.019 (0.46, 2.28)
	Friend	29	11	2.657 (1.39, 5.06)	1.383 (0.54, 3.56)
	Mobilizer	41	12	2.593 (1.36, 4.95)	1.265 (0.49, 3.27)
	Peer educator	10	3	1	1
Reachable					
	Yes	192	63	3.107 (1.98, 4.83)	3.786 (1.50, 9.56)
	No	51	51	1	1
Near to their Home(5 km) (Drop-in center)					
	Yes	193	69	2.573 (1.62, 4.08)	2.179 (1.11, 4.30)**
	No	50	46	1	1
PrEP Knowledge					
	Yes	216	80	3.5 (2.21, 5.56)*	3.27 (1.30, 8.23)**
	No	27	35	1	1

*p < 0.2; **p < 0.05.

## 4 Discussion

The study was conducted to determine the level of acceptance of pre-exposure prophylaxis (PrEP) and associated factors among female sex workers in Addis Ababa. In this study, more than two-thirds (67%) of female sex workers accepted PrEP. This finding is consistent with studies conducted in other regions of sub-Saharan Africa, such as Kampala, Uganda, where 71% of female sex workers accepted PrEP (27). Similarly, in Xinjiang, China, the acceptance rate was 69.29% (28). Additionally, a study in Uganda found a similar PrEP acceptance rate of 66% among female sex workers (18).

However, the current study’s PrEP acceptance rate is significantly lower than the 80.4% reported in Kolkata, India (14), 76.1% in Ho Chi Minh City, Vietnam (29), 92.2% in South-Central Uganda (30),91% in Zambia (18) and another cross-sectional study done in US show high acceptance rate (74%) (31).

This disparity might be due to the reasons for refusal among the 115 (32.1%) participants who did not accept PrEP. The most common reasons for non-acceptance were fear of stigma and discrimination (29.56%), concerns about drug side effects (18%), lack of information about the drug (13.9%), and preference for condoms (12.1%). Additionally, 7.8% believed that God keeps them safe, 6.95% thought they were HIV negative, 6% felt confident in their protective measures, and 5.2% cited other reasons such as dislike of the drug and inconvenience of daily pills. Furthermore, this disparity might also stem from cultural and economic differences in the study areas. The healthcare infrastructure in Ethiopia poses significant barriers to accessing pre-exposure prophylaxis (PrEP), particularly when compared to countries with more integrated and efficient healthcare systems. In nations where access to preventive services is streamlined, individuals are more likely to encounter fewer obstacles when seeking PrEP, thereby fostering higher acceptance rates (32). Furthermore, the level of training that healthcare providers receive regarding PrEP is crucial in influencing acceptance rates. In environments where providers are well-informed and actively engage in discussions about PrEP, patients are likely to feel more comfortable and empowered to consider this preventive option (33). Additionally, the overarching policy environment significantly shapes the effectiveness of health interventions. Countries that implement supportive policies and allocate adequate funding for PrEP programs create conducive environments that facilitate uptake. Conversely, Ethiopia faces various challenges within its policy framework that may impede the effective implementation of PrEP, limiting its availability and accessibility to those at risk (34). These reasons are similar to those reported in a study in Dares Salaam, Tanzania, where 46% of female sex workers were not interested in a daily pill for HIV prevention (35). Similarly, in a high-risk Peruvian population, potential side effects and stigma had the greatest impact on PrEP use (17), while concerns about side effects and low self-perceived risk were the most common reasons for PrEP non-acceptance (36). The leading barrier to PrEP acceptance in the current study was stigma and discrimination (29.56%), which is consistent with findings from other studies (37, 38).

The current research revealed that the acceptance of Pre-Exposure Prophylaxis (PrEP) was significantly and positively associated with both the accessibility of the drug at easily reachable locations and the level of knowledge about PrEP. This finding is consistent with the study conducted by Cornell et al. (39) in South Africa, which demonstrated that increasing the availability of PrEP through community-based distribution points significantly improved uptake among high-risk populations. Similarly, Harrison et al. (40) found that expanding PrEP services to local health centers and community clinics in Kenya resulted in increased PrEP adherence among key populations. Moreover, Harling et al. (35) highlighted in their study in Tanzania that accessibility measures, such as the presence of mobile clinics and local health initiatives, significantly influenced PrEP uptake among female sex workers. Their research emphasized that localized service delivery improved accessibility and consequently led to higher acceptance rates.

Additionally, there was a highly significant difference between individuals who were knowledgeable about PrEP and those who were not. Those who were informed about PrEP were 3.2 times more likely to accept it than those who lacked this knowledge (AOR = 3.27, 95% CI: 1.30–8.23). This finding is consistent with studies from other regions that emphasize the importance of PrEP education. For instance, Cornell et al. (39) found that increasing awareness and understanding of PrEP through educational programs in South Africa significantly improved PrEP uptake among high-risk populations. Similarly, Nabunya et al. (41) demonstrated that community-based educational interventions in Uganda effectively enhanced PrEP knowledge and increased acceptance among high-risk groups. Furthermore, Musa et al. (42) showed that educational interventions in Nigeria also significantly improved PrEP knowledge and acceptance among high-risk individuals. These studies collectively highlight that effective educational strategies are crucial for increasing PrEP acceptance and uptake. Even though the success of PrEP intervention is heavily dependent on the user’s willingness and the compliance with prescribed PrEP regimen lack of awareness and insufficient knowledge regarding this technique by both healthcare providers and subjects are major barriers to its optimal benefit (43–46).

### 4.1 Strengths and limitations of this study

- The study addresses HIV susceptibility among high-risk female sex workers in Addis Ababa, Ethiopia, contributing valuable insights to public health interventions in sub-Saharan Africa.
- Provides a more comprehensive understanding of the factors influencing PrEP acceptance within the target population.
- Given the sensitive nature of the topic (sex work and HIV prevention), participants may be inclined to provide responses they perceive as socially acceptable rather than expressing their true attitudes and behaviors.

## 5 Conclusion

Acceptability of pre-exposure prophylaxis among female sex workers at selected sub-cities (Yeka, Akakikality, and Kirkose) of Addis Ababa Ethiopia is 67.9% which was low. Easily reachability of the area where PrEP is given and female sex workers knowledge about PrEP could be significantly associated with high acceptance. A comprehensive mix of multiple interventions is necessary for the successful implementation of a PrEP program among this population in Addis Ababa.

## Data Availability

The original contributions presented in the study are included in the article/supplementary material, further inquiries can be directed to the corresponding author/s.
